# *QuickStats:* Age-Adjusted Death Rates* for Stroke,^†^ by Region^§^ — National Vital Statistics System, United States, 2001–2021

**DOI:** 10.15585/mmwr.mm7240a5

**Published:** 2023-10-06

**Figure:**
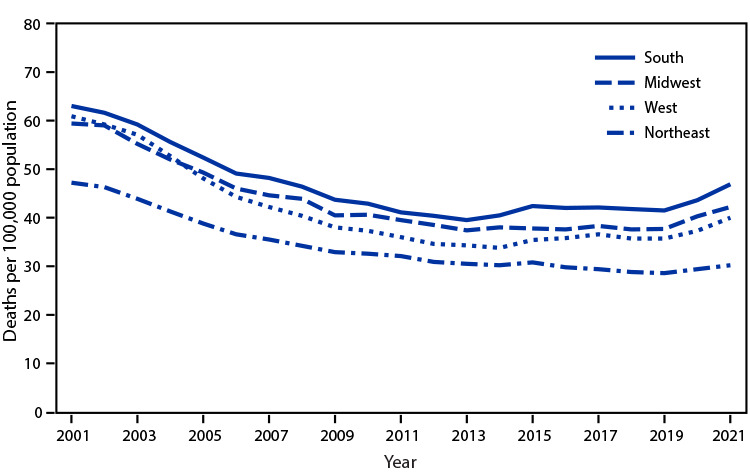
The age-adjusted death rate for stroke declined for all regions from 2001 to 2021. Stroke death rates declined from 2001 through 2013 for persons living in the South (63.0 to 39.5 per 100,000 population) and Midwest (59.4 to 37.4), through 2014 for persons living in the West (60.9 to 33.8), and through 2019 for persons living in the Northeast (47.2 to 28.6). However, rates then increased through 2021 for all regions (South = 46.9, Midwest = 42.2, West = 40.0, and Northeast = 30.2). Despite these later increases, rates in all regions remained lower in 2021 compared with 2001. Throughout the period, stroke death rates were highest in the South and lowest in the Northeast.

**Source:** National Vital Statistics System, Mortality Data, 2001–2021. https://www.cdc.gov/nchs/nvss/deaths.htm

